# Investigational treatments of β-cell failure and replacement

**DOI:** 10.1007/s13340-026-00878-6

**Published:** 2026-02-17

**Authors:** Domenico Accili, Wen Du, Takumi Kitamoto, Wendy McKimpson, Jinsook Son, Hitoshi Watanabe

**Affiliations:** 1 Department of Medicine, Vagelos College of Physicians and Surgeons of Columbia University, New York, NY 10032 USA; 2 School of Biomedical Engineering, Guangzhou Medical University, Guangzhou, 511436 Guangdong People’s Republic of China; 3 Department of Diabetes, Metabolism and Endocrinology, Chiba University Hospital, Chiba, 260-8670 Japan; 4 Department of Endocrinology, Hematology and Gerontology, Graduate School of Medicine, Chiba University, Chiba, 260-8670 Japan; 5 Department of Medicine, Emory University, Atlanta, GA 30322 USA; 6 Division of Endocrinology, Diabetes and Metabolism, Department of Medicine, Weill Center for Metabolic Health, Weill Cornell Medicine, New York, NY 10065 USA

**Keywords:** Diabetes, Type 1, Type 2, Dedifferentiation, Drug development, Enteroendocrine cell

## Abstract

Diabetes is associated with β-cell destruction (Type 1) or functional failure (Type 2). Our research has shown that β-cell failure in Type 2 Diabetes is secondary to the progression of β-cell dedifferentiation. Until recently, it was unclear whether the process was reversible. By analyzing the molecular underpinning of β-cell dedifferentiation, we identified ectopic activation of the enzyme aldehyde dehydrogenase subtype 1A3 (ALDH1A3) as an early marker and effector of the process. Although the signaling pathways by which activation of ALDH1A3 impinges on β-cell function are still to be determined, the enzyme provides a tractable pharmacological target. We have shown that a proprietary, highly potent, and specific ALDH1A3 inhibitor can reverse β-cell dysfunction in animals. Clinical trials of a further version of this compound are being readied. Another area of our interest is the treatment of Type 1 Diabetes by conversion of intestinal epithelial cells into glucose-responsive insulin-producing, β-like cells. We have developed small molecule FoxO1 inhibitors that, when administered orally to diabetic rodents, can lower glycemia and generate insulin-immunoreactive intestinal cells. These cells can also be generated in NOD mice and lead to a restoration of insulin production, demonstrating that they are resistant to autoimmunity. Further preclinical studies are underway to test safety and efficacy of this approach as a Type 1 Diabetes treatment.

## Introduction

Type 2 diabetes (T2D) is characterized by pancreatic β-cell function and mass abnormalities [1]. Clinical features of β-cell failure include limited ability to increase of β-cell mass [2, 3], increased glucagon production and α-cell mass [4], rapid progression in response to even modest elevation of glycemia [5], and time-limited response to different agents utilized in the clinic [6, 7]. In this regard, the key question is arguably whether β-cell failure is reversible [8]. Understanding islet cell dysfunction is critical to design mechanism-based, durable, and safe disease-modifying interventions [9]. But the limited ability to access the endocrine pancreas in vivo, has hindered our ability to functionally interrogate islet cells. To wit, despite intensive, multi-center efforts to standardize procurement and analysis of human islets, there is no consensus on a diabetic gene expression signature [10], let alone on a mechanistic pathway to β-cell failure.

**Fig. 1:**
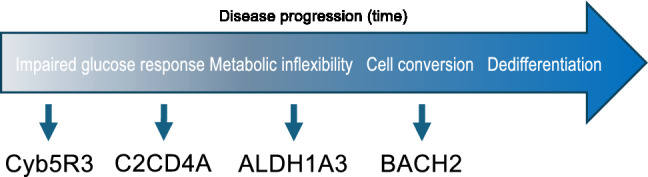
Stages in β-cell failure and potential effectors. We have characterized different phases leading to dedifferentiation. In each stage, we have identified a critical mediator of the process, and in three instances (Cyb5R3, ALDH1A3 and BACH2), we have provided proof-of-principle that their inhibition can relieve or reverse β-cell failure

Our research established that β-cells become gradually dedifferentiated during the progression of T2D (Fig. 1) [11]. We have identified distinct stages in the process of dedifferentiation. During the early phase, β-cells display a characteristic impairment of glucose-induced insulin secretion [12] and activation of “disallowed” genes [13], followed by a metabolic switch that leads to preferential use of lipid-derived acetyl-CoA as a source of mitochondrial oxidative phosphorylation [14–16], and finally by an overt loss of differentiated features [17], accompanied by the activation of a progenitor-like program [17–19]. This process occurs in islets from patients with T2D, as demonstrated by several surveys of islets in individuals of different genetic and ethnic backgrounds [20–22]. Indeed, we suggest that, despite obvious differences, the key underpinnings of β-cell function and failure are preserved across species.

## Discovery of ALDH1A3 as a therapeutic target

Among processes involved in dedifferentiation, activation of expression of the enzyme aldehyde dehydrogenase 1A3 (ALDH1A3) is an early event in the progression of β-cell failure [20, 23, 24]. This is a widely reproduced observation across different models of diabetes in murine and human islets [25–36]. We have investigated its therapeutic potential and mechanism of action in β-cells. Questions raised by our prior work include: (*i*) is ALDH1A3 a marker or an effector of β-cell failure; (*ii*) is ALDH1A3 induction reversible? To answer these questions, we generated mutant alleles in mice to lineage-trace ALDH1A3-expressing cells, and somatically ablate ALDH1A3 in β-cells.

**Fig. 2:**
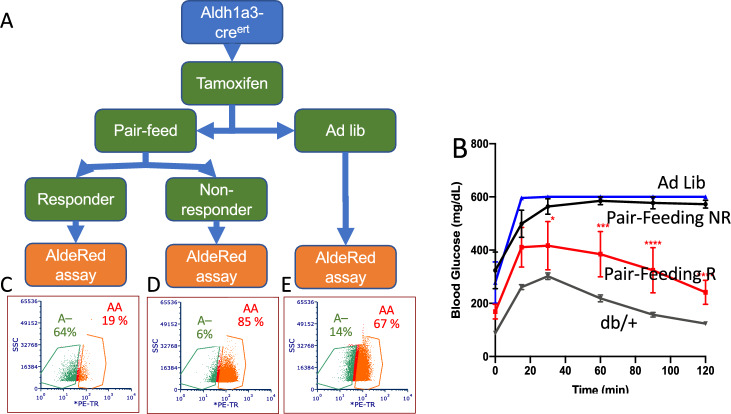
ALDH1A3 in β-cell function. **A** Strategy to lineage-trace ALDH1A3-expressing cells. A tamoxifen pulse is administered to label ALDH1A3-expressing cells. Thereafter, animals are either fed *ad lib*, or pair-fed with *db/* + as controls for 4 weeks. Islet cells are incubated with AldeRed, then sorted based on GFP and red fluorescence. **B** ipGTT in *db/db/Aldh1a3*-YFP mice following pair feeding (n = 6 each sub-group). **C–E** Flow cytometry profiles and relative percentages of recovering (A–) and failing (AA) b-cells following pair feeding

*ALDH1A3 induction is reversible in db/db mice.* We have previously shown that pair-feeding *db/db* mice greatly reduced the number of ALDHA3-expressing β-cells, but we didn’t know if this was due to reversal of β-cell failure, or β-cell death followed by the appearance of new cells [24]. To determine the reversibility of ALDH1A3 induction, we used a dual-labeling strategy: lineage-tracing ALDH1A3-expressing cells with cre reporter alleles, and ongoing enzymatic activity in live cells with AldeRed (Fig. 2A). We labeled cells that at some point in their lives had expressed ALDH1A3 using *Aldh1a3*-cre^ERT^ mice crossed with *db/db;Rosa26*-*lox-STOP-lox-*YFP mice, then treated animals with tamoxifen for one week and pair-fed tamoxifen-treated animals for four weeks. As previously reported [24], at the end of the experiment ~ 2/3 of the pair-fed animals responded with lower fasting glucose and improved GTT, 1/3 didn’t (Fig. 2B). We isolated islets from each group and incubated them with AldeRed, a red fluorescent ALDH1A3 substrate [37], to label cells with ongoing ALDH1A3 activity. As a result, the latter are double-labeled (orange, AA cells, Fig. 2C–E), whereas cells that reverted to ALDH1A3-negative are enzymatically inactive (green A– cells, Fig. 2). In *ad lib*-fed mice, we detected 67% AA and 14% A– cells (red, Fig. 2E). In non-responders to pair-feeding, AA cells increased further to 85%, and A– decreased to 6% (Fig. 2D). In contrast, in responders to pair-feeding with improved glucose metabolism, AA cells decreased to 19%, while A– rose to 64% (Fig. 2C). Thus, improvement of diabetes is associated with a tenfold rise of A– cells, and a fourfold decrease of AA cells. We subjected AA and A– cells to RNAseq and confirmed that *Aldh1a3* was the top increased mRNA in AA cells, and the top decreased in A– cells. This result demonstrates that improvement of β-cell function is associated with reversal of ALDH1A3 activation, whereas worsening β-cell failure is associated with more ALDH1A3-active cells. The data suggest that β-cell failure can be reversed during the dedifferentiation process [38, 39]. Thus, we conclude that β-cell failure is reversible, at least in its initial stages.

**Fig. 3:**
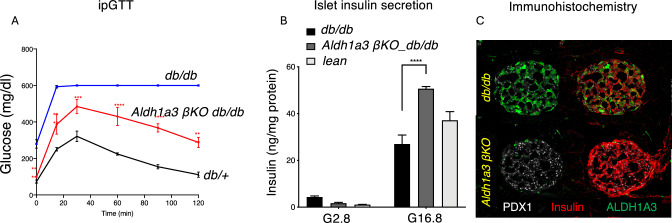
Genetic ablation of ALDH1A3 in β-cells of mice. **A** Intraperitoneal glucose tolerance tests in *db/db*, Aldh1a3 β-cell specific knockout *db/db* and *db/* + mice (n = 6 each group). **B** Glucose-dependent insulin secretion in islets from mice of the same three genotypes. **C** Immunohistochemistry with antibodies against insulin (red), PDX1 (white), and ALDH1A3 (green) in islets from *db/db* and Aldh1a3 β-cell specific knockout mice

*Role of ALDH1A3 in β-cell failure*. To understand whether ALDH1A3 is merely a marker of disease progression or participated in its pathogenesis, we generated β-cell-specific *Aldh1a3* knockout mice [40], and backcrossed them on a *db/db* background. Serial experiments in separate cohorts showed that ALDH1A3 ablation normalized fasting glucose and lowered glucose excursions during IPGTT by ~ 50% compared to *db/db* (Fig. 3A). We purified islets for static incubations to measure insulin secretion. Glucose-dependent insulin secretion from islets increased by 50% compared to *db/db*, and outperformed WT islets (Fig. 3B). In addition, markers of healthy β-cell function, such as insulin and PDX1 were restored following ALDH1A3 ablation (Fig 3C). These data indicate that ALDH1A3 inactivation in β-cells partly protects *db/db* mice against diabetes. Thus, ALDH1A3 activation contributes to β-cell dysfunction, and it’s not just a marker thereof. We also performed experiments in collaboration with Kayothera, using their proprietary ALDH1A3 inhibitor, KTX. When administered to diet-induced diabetic or *db/db* mice, KTX significantly reduced glucose levels, while increasing insulin release in response to glucose in islets from either *db/db* animals or T2D donors. Further variations of these compounds have now completed safety and toxicity testing and will undergo Phase 1 trials in T2D patients in the near future [39].

## Intestinal epithelial cell conversion as a treatment of type 1 diabetes

Restoration of insulin production is the Holy Grail of T1D treatment. We have shown that a subset of intestinal epithelial cells can be reprogrammed to take on a lineage similar to pancreatic β-cells. When FoxO1 is ablated by gene knockout or inhibited by shRNA [41], a subset of enterochromaffin cells convert to β-like-cells that not only make, but also secrete insulin in a glucose dose-dependent fashion [42]. The newly arisen intestinal insulin-producing cells have all the markings of pancreatic β-cells and can take over the function of pancreatic β-cells after the latter have been destroyed by the toxin, streptozotocin, effectively “curing” diabetes in rodents [42]. Unlike iPS-derived β-like cells, enteroendocrine-derived β-like-cells display glucose-dose-dependent insulin release as soon as they arise [42, 43]. These experiments found independent confirmation in work carried out in the Stanger [44] and Zhou laboratories [45]. However, these observations are of limited translational value.

**Fig. 4:**
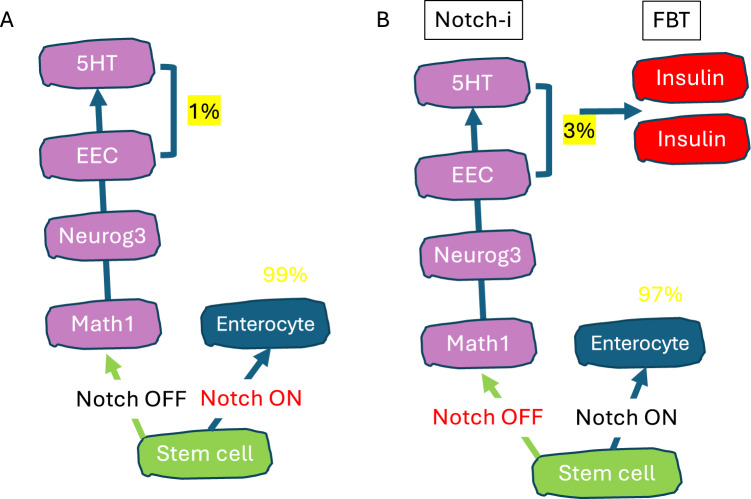
Combined FoxO/Notch inhibition. **A** During intestinal stem cell differentiation, inhibition of Notch signaling leads to activation of Math1, Neurog3, and EEC differentiation. **B** By combining a Notch inhibitor with a FoxO1 inhibitor (FBT), we increased the number of cells primed for conversion to insulin-producing cells (1–3%)

To translate our discovery into a therapeutic approach, we demonstrated that the process is not limited to murine enteroendocrine cells (EEC) but also happens in human iPS-derived gut organoids, as well as primary organoids [41]. We performed lineage tracing experiments to show that different gut epithelial cell types derived from the Neurogenin3-positive progenitor cell, including enterochromaffin (producing serotonin, 5HT), Paneth, and goblet cell lineages can also undergo conversion to the insulin lineage [46]. To carry forward these basic science observations into a clinical development program, we collaborated with the biotech company, Forkhead Biotherapeutics. The company developed novel chemical FoxO1 inhibitors that can selectively modulate FoxO1 target genes and thus mimic the effect of a genetic FoxO1 knockout. We profiled two of their FoxO1 inhibitors (FBT432 and FBT374) and showed that these compounds can convert gut cells in vivo in three different mouse models of insulin-deficient diabetes: STZ-induced, Akita, and NOD mice, the latter of which has an autoimmune pathogenesis [43, 46, 47]. The recovery from diabetes in NOD mice treated with FoxO1 inhibitors also shows that these cells have the potential to escape the autoimmunity of T1D, thus circumventing the need for immunomodulatory or immunosuppressive therapies [46]. Inhibition of the Notch signaling pathway is required during intestinal development to drive cells into the enteroendocrine lineage [48]. Notch and Foxo are mechanistically related, often regulating the same genes in coordination (Fig. 4) [49, 50]. Based on this rationale, we showed that, in addition to monotherapy with FoxO1 inhibitors, combination therapy in which FoxO1 inhibitors are administered with either a Notch inhibitor [43, 47] or with Notch and TGF-β [46] inhibitors can convert gut cells and provide a source of insulin-producing cells that lower glycemia in mice. It should be pointed out that Notch inhibitors have been in the clinic for some time, although their main indication–cancer–didn’t show efficacy.

We sought to optimize methods to induce conversion of EEC and other intestinal epithelial cell types (goblet and Paneth) to insulin-producing, β-like cells. EEC cells account for ~ 1% of the estimated 10^14^–10^15^ gut epithelial cells in the human body [51]. Thus, the number of potentially convertible EEC far exceeds the ~ 10^9^ β-cells in the endocrine pancreas of an average non-diabetic person; hence, successful conversion of only a small fraction of EEC should suffice to achieve a robust therapeutic effect [52]. Unlike slow-turnover pancreatic islet cells, EEC turn over every 3–5 days and regenerate throughout life [53]. The converted gut β-like cells are therefore not subject to chronic stresses posed by autoimmunity or inflammation that are known to induce β-cell failure [54], as gut cells are regularly shed into the lumen and replaced by newly converted cells. This is a key advantage compared to cell replacement therapy, in which surgically implanted β-cells must remain functional in the host for a prolonged period of time to be a therapeutically viable option. The rapid turnover of gut cells also presents a safety advantage if adverse gastrointestinal side effects develop. In the case of T1D, the converted gut β-like cells may be less susceptible to the autoimmunity that kills pancreatic β-cells, since the gut is a more immune-tolerant organ, being constantly exposed to foreign agents such as food and microbiota [55].

## Conclusions

Despite the increased availability of potent and durable anti-diabetic agents as well as glucose monitoring with tethered insulin delivery systems, the number of diabetic patients achieving target HbA1c values has remained stable in the 1999–2018 NHANES survey [56], and gaps remain in T1D treatment [57]. Thus, there remains a need for disease-modifying treatments that can achieve disease stabilization or remission. Our research continues to be focused on these goals.
